# Ropeginterferon alpha-2b targets JAK2V617F-positive polycythemia vera cells in vitro and in vivo

**DOI:** 10.1038/s41408-018-0133-0

**Published:** 2018-10-04

**Authors:** Emmanuelle Verger, Juliette Soret-Dulphy, Nabih Maslah, Lydia Roy, Jerome Rey, Zineb Ghrieb, Robert Kralovics, Heinz Gisslinger, Barbara Grohmann-Izay, Christoph Klade, Christine Chomienne, Stéphane Giraudier, Bruno Cassinat, Jean-Jacques Kiladjian

**Affiliations:** 1 Hôpital Saint-Louis, service de Biologie Cellulaire, Assistance Publique Hôpitaux de Paris, Paris, France; 2 Centre d’Investigations Cliniques, Hôpital Saint-Louis (AP-HP), Paris, France; 3 Hématologie, Hôpital Henri Mondor, Créteil, France; 4 Hématologie, Institut Paoli-Calmettes, Marseille, France; 5 Genetics of hematological disorders, CeMM Research Center for Molecular Medicine of the Austrian Academy of Sciences, Wien, Austria; 6 Department of hematology and blood coagulation, Medical University of Vienna, Wien, Austria; 7 AOP Orphan Pharmaceuticals Aktiengesellschaft, Wien, Austria; 8 INSERM UMRS_1131, Institut Universitaire d’Hématologie, Hôpital Saint-Louis, Paris, France; 9 Université Paris Diderot, Paris, France

## Abstract

Polycythemia vera is characterized by the acquisition of the JAK2V617F mutation. Recommended treatments include hydroxyurea and interferon-alpha. Several groups have reported a reduction in the JAK2 mutant allele burden in interferon-treated patients, but significance of this observation is questioned. We characterized the activity of ropeginterferon alpha-2b, a novel form of interferon-alpha recently shown to be safe and efficacious in polycythemia vera. Ropeginterferon was able to inhibit the proliferation of the HEL, UKE-1, and UT-7 JAK2-mutant cell lines while sparing JAK2-wild-type UT-7 and normal CD34+ cells growth. In vitro treatment of erythroid progenitors derived from PV patients showed that ropeginterferon could considerably inhibit the growth of endogenous erythroid colonies, a hallmark of polycythemia vera. Finally, we could study in sequential samples the clonal architecture of erythroid progenitors derived from patients included in a randomized study comparing hydroxyurea to ropeginterferon. After 1 year of treatment with ropeginterferon, the ratio of *JAK2*-mutated to wild-type colonies grown from bone marrow progenitors was reduced by 64%, compared to 25% in patients receiving hydroxyurea. This study shows that ropeginterferon has a potent targeted activity against *JAK2*-mutant cells and is able to drastically reduce the proportion of malignant progenitors in patients treated with this drug.

## Introduction

Polycythemia vera (PV) is characterized by a deregulated erythropoiesis related to the presence of the JAK2^V617F^ mutation^1^, which induces a constitutive activation of the JAK/STAT intracellular signaling. More recently, additional mutations in various genes have been discovered, including mutations in genes affecting the epigenome (*TET2*, *ASXL1*, *DNMT3a*, or *EZH2)*, or contributing to transformation to acute leukemia (*NRAS*, *KRAS*, *TP53*, or *IDH1/2)*^2,3^. The presence of such additional mutations may also influence the response to therapy^4^. Current first-line recommended cytoreductive treatment of PV include hydroxyurea (HU) or interferon-alfa (IFNα)^5–7^. The clinical efficacy of IFNα has been reported since 30 years^8^ and was improved with the development of pegylated forms^9,10^. Furthermore, we and others have observed significant reductions of the JAK2^V617F^ allele burden (%JAK2^V617F^) in IFNα-treated patients^11,12^ suggesting that IFNα is able to specifically target the malignant clone. The mechanism of action of IFNα in myeloproliferative neoplasms is not clearly elucidated, but several studies confirmed a targeted effect against JAK2^V617F^ mutant clones in both patients^13^ and animal models^14,15^. In clinical practice, a significant proportion of patients still experience adverse events leading to treatment discontinuation with current formulations of IFNα^16^. RopegInterferon alpha-2b (Ropeg) is a long-acting pegylated-IFNα-2b, recently shown to be safe and well tolerated in phase 1–2 studies in PV patients^17,18^. Both hematological and molecular responses have been reported in a phase 2 trial including 51 patients.

In this study, we characterized Ropeg activity against JAK2^V617F^ mutated cells. We tested this new drug in vitro against *JAK2*-mutant and wild-type cell lines and patients’ primary cells. We could also assess the impact of HU and Ropeg treatments in vivo by sequential studies of bone marrow (BM) progenitors of PV patients treated for 12 months with both drugs in a prospective, randomized study.

## Patients and methods

### Cell lines and reagents

Ropeg (provided by AOP Orphan) was used at two different concentrations consistent with assumed exposure in patients (0.5 and 2 µg/ml), and commercially available standard recombinant interferon-alpha-2a (rIFNα-2a) (Roferon, Roche) was used at 700 U/ml as control. The JAK2^V617F^ positive HEL and UKE-1 cell lines were grown in RPMI + 10% FBS + 1% Glutamax, and IMDM + 10% horse serum + 10% FBS and 1% Glutamax, respectively. We also used the megakaryoblastic leukemia UT-7 cell line expressing the EPO receptor in which either a wild type or a JAK2^V617F^ mutant JAK2 were retrovirally transduced. These cells were cultured in DMEM + 10% FBS + 2 U/mL EPO. Drug efficacy was assessed by counting live cells after Trypan blue staining. All cell lines were tested and found negative for mycoplasma contamination.

### Patients

Normal hematopoietic progenitors derived from cord blood and primary cells from PV patients obtained after informed consent were studied in clonogenic assays with or without EPO according to manufacturer’s instructions (Methocult, Stemcell technologies©). The study was approved by the local ethics committee (IRB0006477). The presence of endogenous erythroid colonies (EECs) was determined in cultures without erythropoietin (EPO). Genotyping of the colonies (at least 60 colonies were tested in each condition) was performed by picking, extracting the DNA and testing for the presence of JAK2^V617F^ mutation using the JAK2 Mutascreen kit (Qiagen©). We studied untreated patients and patients included in the PROUD-PV phase 3 clinical trial (NCT01949805) randomly treated with Ropeg or HU. A total of 13 patients have been included in the PROUD-PV study in France for which BM samples were sent to our laboratory before the initiation of treatment and after 1 year. Informed consent was obtained from all subjects.

### Mutant allele burden assessment

The allelic frequency of the JAK2^V617F^ mutation was assessed on DNA extracted from whole blood (QiaAmp DNA blood mini kit, Qiagen) using the JAK2 MutaQuant kit (Qiagen) according to the manufacturer’s instruction. Search for additional mutations was performed using a targeted next generation sequencing (NGS) assay as previously described^4^.

## Results

**Fig. 1:**
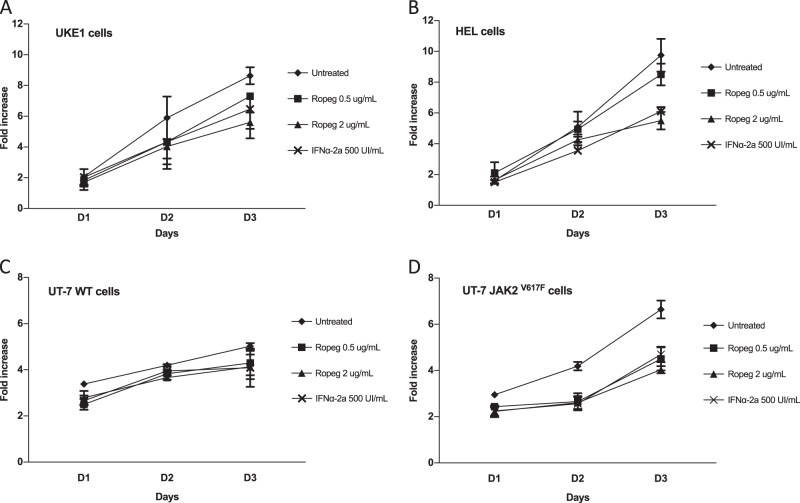
Antiproliferative effect of Ropeg in MPN-derived human cell lines. **A** and **B** The JAK2^V617F^ positive UKE1 and HEL cell lines. **C** and **D** The UT-7 cell line expressing a wild type or a mutant form of JAK2. Cells were treated with the indicated drugs and the living cells were counted every day. Results are expressed as the fold increase compared to day 0

We first intended to evaluate the action of Ropeg on the proliferation of JAK2^V617F^-mutated cell lines. In both HEL and UKE-1 cell lines Ropeg exhibited a dose-dependent anti-proliferative effect, comparable to that of standard rIFNα-2a (Fig. 1A, B; Supplementary figure 1). Ropeg at 0.5 µg/ml and 2 µg/ml induced 9%, and 41% inhibition of HEL cells proliferation, and 18 and 35% inhibition of UKE-1 cells proliferation, respectively (compared to 38%, and 36% inhibition of HEL, and UKE-1 cells proliferation, respectively, with rIFNα-2a). To test for a more-specific action of Ropeg against the JAK2^V617F^ mutant form compared to JAK2 wild type, we used the UT-7 model in which both forms of the *JAK2* gene have been transduced. We observed a modest impact of AOP treatment on the JAK2 wild-type UT-7 cells proliferation (23% of inhibition at day 3) while a marked decrease in the proliferation of JAK2^V617F^ positive cells was observed (40% reduction at day 3 with 2 µg/ml) (Fig. 1C, D; Supplementary Figure 1).

**Fig. 2:**
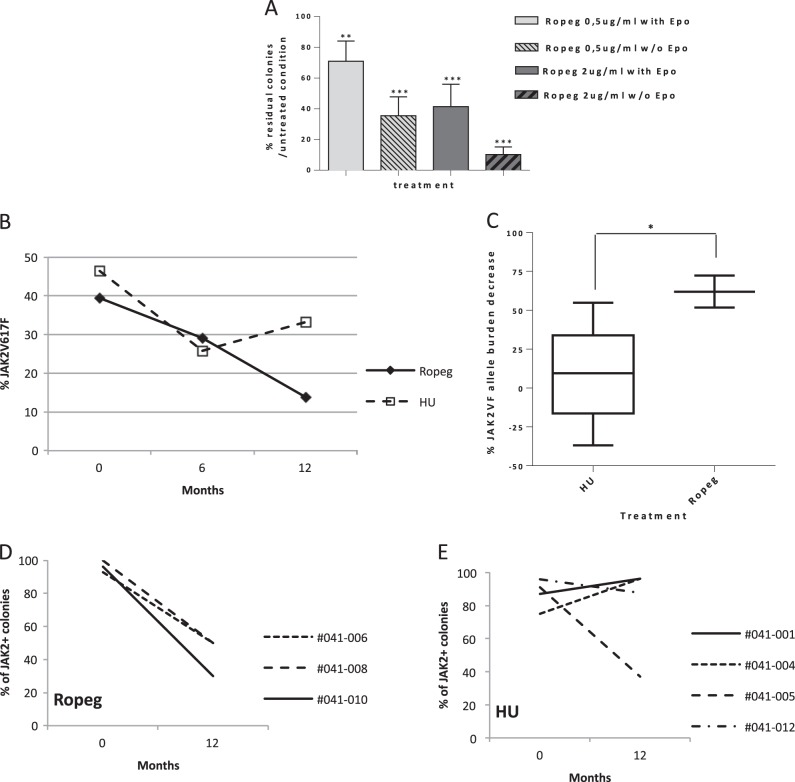
Targeted inhibition of JAK2^V617F^ progenitors in vitro and in vivo **A** Clonogenic assays on primary peripheral blood mononuclear cells from 4 PV patients. Median percentages and standard deviations of residual erythroid colonies in treated conditions compared to untreated are presented. **B** and **C** JAK2^V617F^ allele burden evolution in patients included in the PROUD-PV trial in France. **B** median of the %JAK2V617F in HU (*N* = 5) or Ropeg (*N* = 3) treatment arms. **C** Comparison of the % reduction of JAK2V617F allele burden in patients after 1 year of treatment in each arm. Median and standard deviations in both groups of treatment are represented. (*p < 0.02; unpaired *t*-test). **D** and **E** Clonogenic assays performed on bone marrow samples from patients included in the PROUD-PV trial in France. Percentages of JAK2^V617F^ positive erythroid colonies before and after 1 year of treatment in patients treated with Ropeg or HU. UPN of each individual patient is given

We then studied the impact of an in vitro treatment with Ropeg on the clonogenic potential of erythroid progenitors derived from four untreated PV patients. Ropeg reduced the numbers of colonies grown with or without EPO in all samples. The mean reduction of the numbers of EPO-stimulated colonies was 29 and 59% with Ropeg at 0.5 µg/ml, and 2 µg/ml, respectively. A more striking effect was observed on EECs, the number of colonies being reduced by 90% with Ropeg 2 µg/ml (Fig. 2A). In contrast, Ropeg didnot significantly change clonogenic properties of normal (JAK2 wild type) hematopoietic progenitors isolated from three different cord blood samples (Supplementary figure 2), suggesting that Ropeg inhibits JAK2-mutated hematopoietic progenitors while sparing wild-type cells. To confirm this hypothesis we studied the *JAK2* mutational status at the clonogenic level by genotyping individual colonies grown after in vitro treatment with Ropeg. Single colonies from the 4 PV patients were picked (at least 60 colonies per condition) and genotyped. The proportion of mutant colonies was reduced in every patient (Supplementary figure 3) with a median twofold increase in the ratio of wild type to mutant colonies. In addition, in two patients with both homozygous and heterozygous JAK2^V617F^ colonies, eradication of homozygous colonies was achieved with Ropeg, suggesting that JAK2^V617F^ homozygous progenitors are more sensitive to Ropeg, in agreement with previous findings in MPN patients treated with pegylated-IFNα−2a.

In addition to these in vitro studies, we took advantage of the PROUD-PV randomized study to search for any correlation between the evolution of the %JAK2^V617F^ in peripheral blood and the impact of therapy on malignant clones assessed by clonogenic assays on BM progenitors. Thirteen patients were included in this study in France, who accepted to participate in an ancillary study of the evolution of their BM progenitors before and after 12 months of treatment in both randomization arms. In this group of patients the mean %JAK2^V617F^ at baseline were 39.4% and 46.5% in the Ropeg and HU arms, respectively. Median %JAK2 ^V617F^ was reduced to 29% and 25.8% after 6 months, and to 13.8% and 33.2% after 12 months (Fig. 2B) of treatment with Ropeg and HU, respectively. These data suggested a sustained activity of Ropeg, but not of HU, against JAK2^V617F^ mutant cells. Indeed, we observed a difference between the two drugs considering the median reduction of the %JAK2^V617F^ (*p* < 0.02) and the responses were more homogeneous in Ropeg compared to HU-treated patients (Fig. 2C).

To better assess the impact of both drugs on *JAK2*-mutated cells in vivo we performed clonogenic assays on BM mononuclear cells taken before treatment initiation and after 1 year in order to analyze the changes in clonal architecture. As expected in PV, we observed the presence of EECs in every patient. The ratio of *JAK2*-mutant to *JAK2* wild-type colonies decreased in all of the three patients treated with Ropeg (Fig. 2D) while it decreased in only one out of four patients receiving HU (Fig. 2E). Of note, the poorer clonal response measured by %JAK2^V617F^ in the HU arm wasn't explained by the presence of additional mutations. Using targeted NGS^4^, we could detect additional mutations in only two patients: one *TET2* mutation (p.S393Lfs*34; VAF 27%) in a patient randomized in the HU arm, and one *DNMT3A* mutation (p.S837*; VAF 7%) in a patient included in the Ropeg arm. Interestingly, the only patient in whom the percentage of JAK2-mutant colonies decreased during HU therapy was the patient with concomitant *JAK2* and *TET2* mutations.

## Discussion

IFNα is a cytokine with a wide range of biological properties including antitumor activity used for decades to treat several types of cancers like melanoma, renal cancer, and hematological malignancies, including MPNs^9^. In this study, we assessed the ability of a new form of IFNα to specifically target JAK2-mutant cells. We first showed that Ropeg has an antiproliferative effect on JAK2^V617F^ mutant cell lines similar to that of standard recombinant IFNα. In the UT-7 cell line model we could further show that Ropeg has a twofold greater inhibitory effect against JAK2^V617F^-mutated cells compared to their wild-type counterpart. When tested in cultures of primary hematopoietic progenitors derived from PV patients, Ropeg showed an important inhibitory effect on the growth of erythroid colonies, when it had no particular impact on the growth of erythroid colonies when tested at therapeutic concentrations against normal JAK2-wild-type progenitors. Altogether, these results suggest that Ropeg preferentially targets progenitors harboring the JAK2^V617F^ mutation while sparing wild type cells.

In the subset of patients included in France in the PROUD-PV trial, we observed deeper and more prolonged molecular responses (assessed by the reduction of %JAK2^V617F^ in peripheral blood) in patients randomly assigned to Ropeg versus those receiving HU. In chronic myeloid leukemia, achievement of molecular remission has been clearly associated with better clinical outcome and possible treatment free remissions after discontinuation of tyrosine-kinase inhibitor treatment^19^. Such correlation between reduction of the JAK2 mutant allele burden in PV with IFNα and clinically relevant outcomes has not been demonstrated yet. To better understand the biological significance of sustained circulating mutant allele burden reduction, we studied the evolution of clonal architecture of hematopoietic progenitors in PV patients randomly treated with IFNα or HU in the PROUD-PV study. Clonal studies of bone marrow progenitors of these patients showed that after 12 months of treatment with IFNα the proportion of wild type to mutant *JAK2* colonies was clearly increased, an effect not seen in patients receiving HU. This is to our knowledge the first evidence suggesting that a sustained decrease in the circulating JAK2^V617F^ allele burden could reflect a diminution of the proportion of malignant progenitors in the bone marrow. In that perspective, such results suggest that achievement of deep molecular response in PV could result in the exhaustion of MPN progenitors and open the way for safe cytoreductive treatment discontinuation as currently proposed in CML patients achieving deep molecular response.

Altogether, these preliminary results obtained in a small subset of patients included in the PROUD-PV study must be confirmed in larger numbers, but they suggest that IFNα could be a good candidate for achieving long-term eradication of *JAK2*-mutant clones and restoring normal hematopoiesis in PV patients.

## Electronic supplementary material

Supplementary Figure 1

Supplementary Figure 2

Supplementary Figure 3

## Data Availability

The datasets used and/or analyzed during the current study are available from the corresponding author on reasonable request.
