# Supplementary material for: Ropeginterferon alpha-2b targets JAK2V617F-positive polycythemia vera cells in vitro and in vivo
Source: Blood Cancer J. 2018 Oct 4;8(10):94. doi: 10.1038/s41408-018-0133-0 (PMC6172224; doi:10.1038/s41408-018-0133-0)
Supplement: Supplementary file 1 — Supplementary Figure 1 [file 41408_2018_133_MOESM1_ESM.pdf]

Supplementary Figure 1

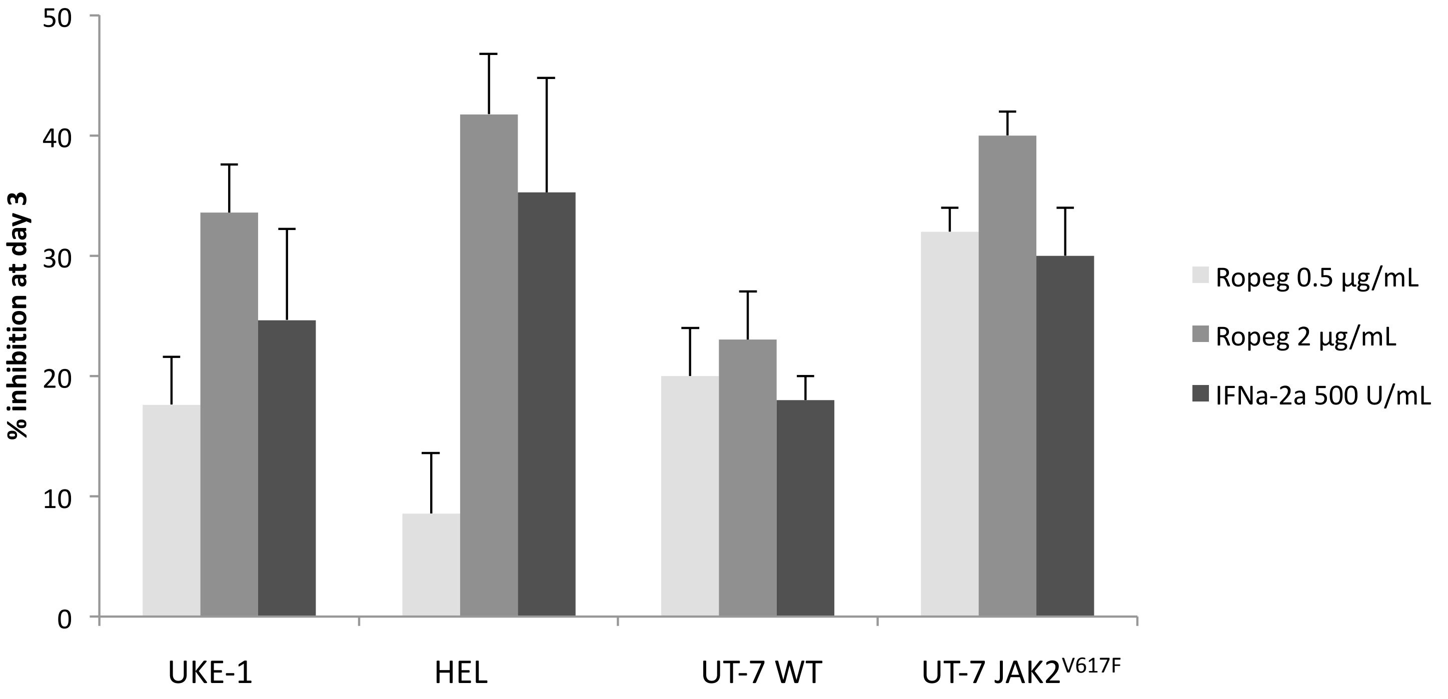

Supplementary Figure 1: Antiproliferative action of Ropeginterferon alpha-2b.

MPN derived cell lines were treated with the indicated drugs and the living cells were counted at day 3. The figure presents the percentages of growth inhibition compared to untreated condition for each cell line.
