# Supplementary material for: Ropeginterferon alpha-2b targets JAK2V617F-positive polycythemia vera cells in vitro and in vivo
Source: Blood Cancer J. 2018 Oct 4;8(10):94. doi: 10.1038/s41408-018-0133-0 (PMC6172224; doi:10.1038/s41408-018-0133-0)
Supplement: Supplementary file 2 — Supplementary Figure 2 [file 41408_2018_133_MOESM2_ESM.pdf]

Supplementary Figure 2

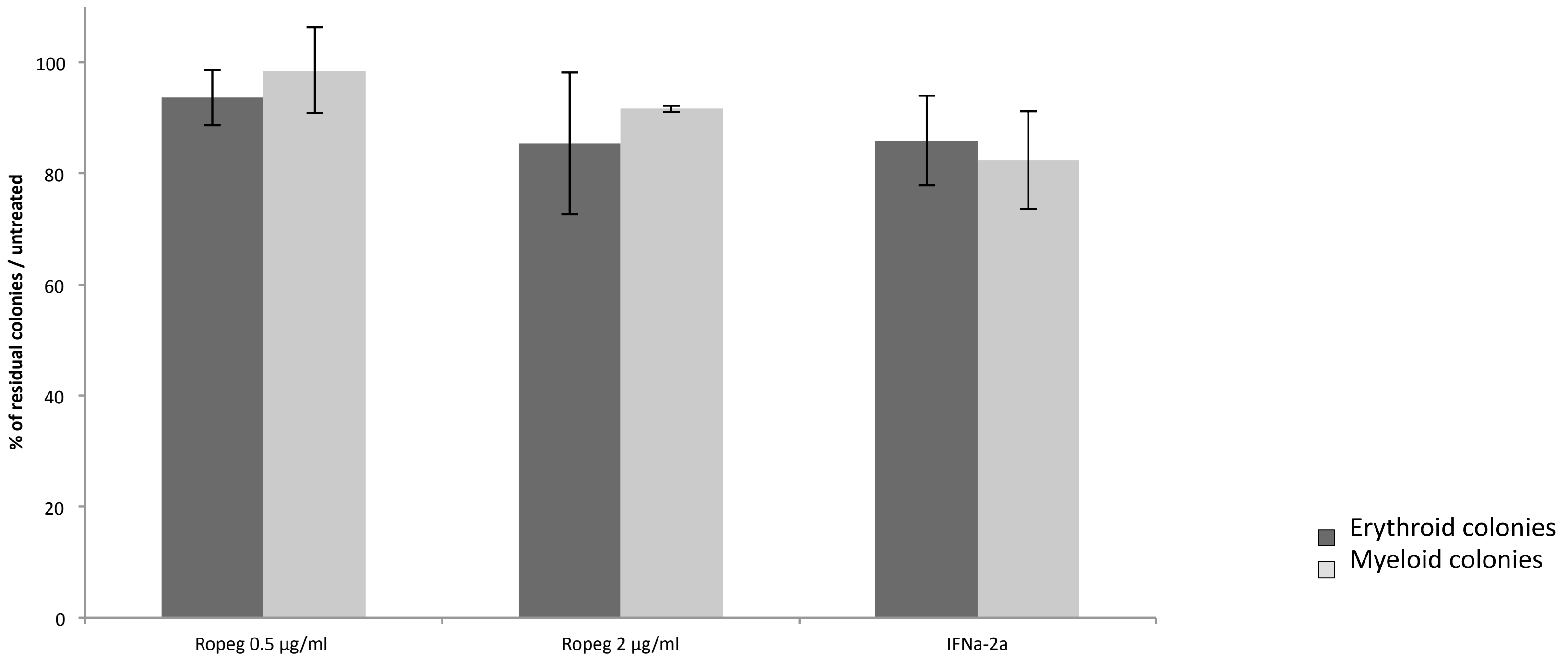

Supplementary Figure 2: Clonogenic assays on primary progenitors from cord blood.

Results obtained from 3 different cord blood samples are presented as the percentages of residual colonies in treated conditions compared to untreated.
