# Supplementary material for: Ropeginterferon alpha-2b targets JAK2V617F-positive polycythemia vera cells in vitro and in vivo
Source: Blood Cancer J. 2018 Oct 4;8(10):94. doi: 10.1038/s41408-018-0133-0 (PMC6172224; doi:10.1038/s41408-018-0133-0)
Supplement: Supplementary file 3 — Supplementary Figure 3 [file 41408_2018_133_MOESM3_ESM.pdf]

Supplementary Figure 3

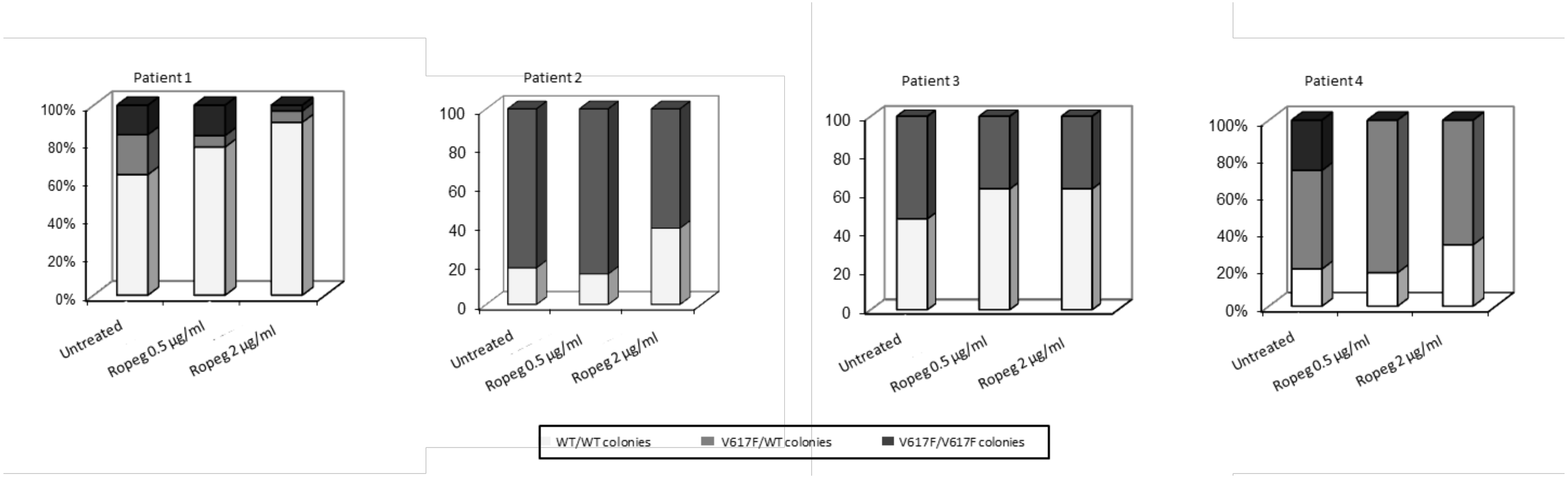

Supplementary Figure 3: Genotype of colonies grown in clonogenic assays on primary peripheral blood mononuclear cells from 4 PV patients.

Results are expressed as the percentages of each JAK2 genotype among the colonies tested (a minimum of 60 colonies were sequenced for each patient).
